# Unexpected outcome from Trousseau syndrome

**DOI:** 10.1186/1471-2482-11-1

**Published:** 2011-01-06

**Authors:** Sri G Thrumurthy, Abeyratne HMP Anuruddha, Merrenna IM De Zoysa, Dharmabandhu N Samarasekera

**Affiliations:** 1 University Surgical Unit, National Hospital of Sri Lanka, Colombo 10, Sri Lanka; 2 Department of General Surgery, Royal Preston Hospital, Lancashire Teaching Hospitals NHS Foundation Trust, Preston, PR2 9HT, UK

## Abstract

**Background:**

Unprovoked superficial thrombophlebitis and subsequent venous thromboembolism are well-described albeit rare presenting features of advanced visceral malignancy that often manifest too late for curative intervention to be beneficial.

**Case Presentation:**

We present the first reported case of early gastric carcinoma presenting with these paraneoplastic phenomena in an otherwise healthy farmer. The early presentation allowed for a curative partial gastrectomy, which itself was complicated by the presence of a deep vein thrombosis extending into the inferior vena cava. Fortunately, stabilization of the clot allowed for surgery to proceed without the need for a caval filter. The patient was referred for adjuvant chemotherapy and has since made an excellent recovery.

**Conclusions:**

This case provides new evidence for the presentation of superficial thrombophlebitis in early gastric carcinoma and the potential for curative surgery in such instances. A high index of suspicion and a prompt diagnostic workup are essential for timely planning and execution of surgery in these early albeit rare presentations.

## Background

Unlike the well-established association of spontaneous venous thromboembolism (VTE) with occult neoplasia, single episodes of unprovoked superficial thrombophlebitis have been suggested not to be associated with malignancy [1]. In contrast, the historically significant Trousseau syndrome (i.e. migratory thrombophlebitis) is a well-described association of advanced malignancy - particularly of pancreatic and pulmonary tumours [2]. The mechanism of this paraneoplastic phenomenon is unknown but has been linked to the late stage of these tumours at presentation, which in all reported cases to date, has precluded any form of curative intervention in these patients [3,4].

## Case presentation

A 41-year-old farmer with no significant past medical history presented to Casualty with acute, severe pain over the medial aspect of his right leg and thigh. Examination revealed exquisitely tender cord-like thickening along the distribution of the long saphenous vein. The patient firmly denied external trauma but an infective cause of the inflammation was presumed 'until proven otherwise'.

Intravenous penicillin and flucloxacillin were administered but the inflammation persisted for a further three days. During this time, routine blood tests revealed a microcytic anemia (hemoglobin 9.3 g/dL, mean corpuscular volume 70 fL) of unclear etiology, suggesting the need for an esophagogastroduodenoscopy (EGD) to exclude upper gastrointestinal tract bleeding. Concurrently, acute swelling of his right calf prompted a duplex ultrasound scan (USS), which revealed a deep vein thrombosis (DVT) with vague suggestion of extension up to the iliac veins. The patient was therefore switched from his prophylactic regimen of low molecular weight heparin (LMWH; 5000 units of subcutaneous dalteparin sodium, once daily) to a therapeutic regimen (15000 units, once daily).

**Figure 1:**
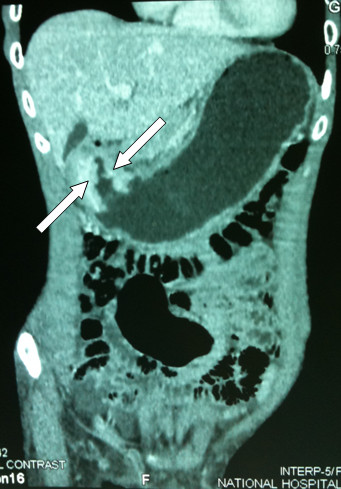
**Contrast computed tomography reconstruction illustrating pyloric tumour (arrows) with consequent gastric outlet obstruction**. No regional lymphadenopathy or distant metastases were noted.

**Figure 2:**
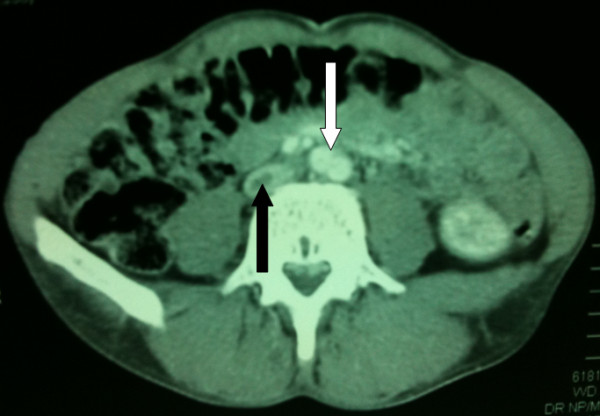
**Contrast computed tomography of the pelvis demonstrating thrombosis (black arrow) within the lower inferior vena cava, just before the abdominal aortic bifurcation (white arrow)**.

The EGD unexpectedly revealed an annular growth around the pylorus partially obstructing the gastric outlet. Biopsy at endoscopy revealed a primary gastric adenocarcinoma. A contrast CT scan demonstrated mucosal confinement of the tumour with no evidence of regional lymph node involvement or distant metastasis (Figure 1). In addition, the CT confirmed thrombosis within the right femoral vein with extension into the lower inferior vena cava (IVC) to just below the renal veins (Figure 2), as previously suggested on duplex USS. Clinical signs of an obstructed IVC were also apparent in the form of dilated superficial lower abdominal veins and a positive Harvey's sign. Nevertheless, the normal renal function and absence of microscopic haematuria supported non-involvement of the renal veins by the DVT.

In anticipation of surgery, anticoagulation was maintained with therapeutic-dose LMWH. A vascular surgical consult advised against the placement of an IVC filter as the relatively prolonged confinement of the DVT and therapeutic heparinization made further extension or dislodgement unlikely. A partial gastrectomy was performed with added precaution taken during dissection around the IVC to avoid thrombus dislodgement. Histology of the resected specimen confirmed a diffuse-type adenocarcinoma with marked acute and chronic inflammation but no lymph node involvement (i.e. T_2_N_0_M_X _or Stage I disease). The patient was subsequently referred for adjuvant chemotherapy and at 6-month follow-up, displayed no signs or symptoms of recurrent disease.

## Conclusions

Although the natural progression of our patient's superficial thrombophlebitis cannot be extrapolated (i.e. to ascertain if it was truly isolated or migratory), this is nonetheless the first reported case of early gastric cancer presenting with this phenomenon and subsequently amenable to curative surgery. The other significant feature of our case was the development of DVT, which is similarly unusual in the early stages of malignancy [5], and occurred despite prophylactic heparinization at admission.

The propagation of DVT to the IVC initially posed a challenge to the surgical management of our patient's gastric carcinoma, as it was unclear whether the risks of deploying a caval filter were warranted prior to major surgery. A recent prospective study by Birkmeyer et al involving 6376 patients revealed that prophylactic IVC filters for gastric surgery did not reduce the risk of pulmonary embolism (PE) but resulted in filter-related complications such as filter migration (e.g. into the heart or pulmonary arteries), insertion-site thrombosis and IVC thrombosis [6]. A study by Deguchi et al similarly demonstrated that the addition of IVC filters to anticoagulant therapy did not significantly reduce the risks of perioperative patients with VTE prior to surgery [7]. It also described occurrences such as major filter migration, persistent attached thrombosis and connection failure. Apart from filter dislocation, complications such as catheter fracture, catheter-related infection and critical IVC occlusion by massive thrombus post-tumour resection (i.e. necessitating open IVC ligation) have also been reported with the use of temporary filters [8]. Nevertheless, it is recommended that when the use of such filters is imperative, they should be removed as soon as the risk of VTE has passed [9].

We were reminded that proximal propagation of stable DVTs after two weeks was highly unlikely, thereby negating the risks of filter insertion. The evidence supporting this includes a study by MacDonald et al, in which 91% of all DVT propagation occurred within 2 weeks of initial diagnosis, leading to the authors' suggestion that follow-up after 2 weeks may be unnecessary [10]. Similarly, Masuda et al suggested a low likelihood of clot propagation after the first 2 weeks from DVT diagnosis, suggesting serial duplex imaging weekly for the first 2 weeks, with an optional repeat scan at 4 weeks to confirm clot lysis [11]. In slight contrast, the most recent evidence by Lautz et al demonstrated that 43% of DVT propagation (or PE) occurred within the initial 2 weeks; and 71% within the first 3 months [12]. In our case, clinical evidence (e.g. lack of haematuria or altered urine output which may have indicated clot extension to renal veins) and contrast CT imaging added to the reassurance of clot stability.

The stability of our patient's thrombus is very likely attributable to the full therapeutic heparinization administered upon DVT diagnosis, the efficacy of which has been proven on many occasions. A study of DVT outcomes by Gillet et al showed no VTE extension or recurrence within the therapeutic anticoagulation period of 1 month, and also within the subsequent 2 months [13]. Schwarz et al demonstrated a DVT propagation rate of 0% (i.e. despite a recurrence rate of 1.9% at 3 weeks) with therapeutic heparinization and compression therapy for 10 days, versus a 25% rate of DVT progression with compression alone (P = 0.0002) [14]. In support of this, Lautz's study showed a VTE recurrence rate of 30% in patients receiving no anticoagulation, 27% in those receiving only prophylactic anticoagulation, and 12% in those receiving therapeutic anticoagulation (P = 0.0003). Together with a previous history of VTE, lack of therapeutic anticoagulation was shown to be an independent predictor of VTE recurrence. Even during follow-up, therapeutic anticoagulation resulted in a DVT resolution rate of 61.2%, compared to 40.0% for prophylactic anticoagulation and 41.0% for no anticoagulation (P = 0.003) [12]. Considering our patient's thrombophlebitic presentation, his initiation upon prophylactic LMWH may have been an oversight of the admitting medical team. Nonetheless, the degree to which he would have benefited from therapeutic heparinization during the first three days of admission remains unknown.

In conclusion, this case provides new evidence for the presentation of superficial thrombophlebitis in early gastric carcinoma and the potential for curative surgery in such instances. A high index of suspicion and a prompt diagnostic workup are essential for timely planning and execution of surgery in these early albeit rare presentations. For resectable malignancy associated with DVT extending up to the IVC, we support a conservative approach (i.e. therapeutic heparinization) until surgery, provided that surveillance can demonstrate adequate clot stabilization over two weeks. Invariably, if DVT occurs on a suspected background of malignancy, therapeutic-dose heparin should be used as the sole means of anticoagulation until the need for surgery is excluded (or surgery is performed), before warfarin is commenced.

## Consent

Written informed consent was obtained from the patient for publication of this case report and any accompanying images. A copy of the written consent is available for review by the Editor-in-Chief of this journal.

## Competing interests

The authors declare that they have no competing interests.

## Authors' contributions

MIMdZ and DNS were responsible for delivering patient care. SGT and AHMP contributed equally towards to drafting of the manuscript while MIMdZ and DNS provided overall supervision and edited the final version of the manuscript. All authors have read and approved the final manuscript.

## Pre-publication history

The pre-publication history for this paper can be accessed here:

http://www.biomedcentral.com/1471-2482/11/1/prepub
